# First-line therapy with palbociclib in patients with advanced HR^+^/HER2^−^ breast cancer: The real-life study PALBOSPAIN

**DOI:** 10.1007/s10549-024-07287-w

**Published:** 2024-04-01

**Authors:** N. Martínez-Jañez, M. Bellet Ezquerra, L. M. Manso Sanchez, F. Henao Carrasco, A. Anton Torres, S. Morales, P. Tolosa Ortega, V. L. Obadia Gil, T. Sampedro, R. Andrés Conejero, L. Calvo-Martinez, E. Galve-Calvo, R. López, F. Ayala de la Pena, S. Lopez-Tarruella, B. A. Hernando Fernandez de Araguiz, L. Boronat Ruiz, T. Martos Cardenas, J. I. Chacon, F. Moreno Antón

**Affiliations:** 1 Medical Oncology Department, Ramon y Cajal University Hospital, Hospital Ramón y Cajal, Ctra. de Colmenar Viejo km. 9,100, Madrid, 28034 Spain; 2 Oncology Department, Vall d’Hebron Institute of Oncology (VHIO)-Cellex Center, Barcelona, Spain; 3 Medical Oncology Department, Hospital Universitario 12 de Octubre, Madrid, Spain; 4 Oncology, Hospital Universitario Virgen Macarena, Seville, Spain; 5 Medical Oncology Department, Hospital Universitario Miguel Servet, Zaragoza, Spain; 6 Medical Oncology Department, Hospital Arnau de Vilanova - Lleida, Alpicat, Spain; 7 Breast Cancer Unit, ICO - Institut Català d’Oncologia l’Hospitalet (Hospital Duran i Reynals) L’Hospitalet De, Llobregat, Spain; 8 Medical Oncology Department, Hospital Universitario de Cabuenes, Gijón, Spain; 9 Medical Oncology Department, Hospital Clinico Universitario Lozano Blesa, Zaragoza, Spain; 10 Medical Oncology Department, CHUAC - Complexo Hospitalario Universitario A Coruña, A Coruña, Spain; 11 Medical Oncology Department, Hospital Universitario de Basurto, Bilbao, Spain; 12 Servicio de Oncología Médica y Grupo de Oncología Médica Traslacional, Hospital Clínico Universitario e Instituto de Investigación Sanitaria-CIBERONC, Santiago de Compostela, Spain; 13 Medical Oncology Department, Hospital General Universitario Morales Meseguer, Murcia, Spain; 14 Medical Oncology Service, Hospital General Universitario Gregorio Marañón, Instituto de Investigación Sanitaria Gregorio Marañón (IiSGM), CIBERONC, Geicam, Universidad Complutense, Madrid, Spain; 15 Medical Oncology Department, Complejo Hospitalario de Burgos -Hospital General Yagüe, Burgos, Spain; 16 Oncology Department, Hospital de la Santa Creu i Sant Pau, Barcelona, Spain; 17 Medical Oncology Department, Hospital del Mar - Parc de Salut Mar, Barcelona, Spain; 18 Medical Oncology Department, Hospital Virgen de la Salud, Toledo, Spain; 19 Medical Oncology Department, Hospital Clínico Universitario San Carlos, Madrid, Spain

**Keywords:** HR^+^/HER2^−^, Advanced breast cancer, Progression-free survival, Overall survival, Palbociclib, First-line treatment

## Abstract

**Purpose:**

To evaluate the efficacy and safety of first-line therapy with palbociclib in a Spanish cohort treated after palbociclib approval.

**Methods:**

PALBOSPAIN is an observational, retrospective, multicenter study evaluating real-world patterns and outcomes with 1 L palbociclib in men and women (any menopausal status) with advanced HR^+^/HER2– BC diagnosed between November 2017 and November 2019. The primary endpoint was real-world progression-free survival (rw-PFS). Secondary endpoints included overall survival (OS), the real-world response rate (rw-RR), the clinical benefit rate, palbociclib dose reduction, and safety.

**Results:**

A total of 762 patients were included. The median rw-PFS and OS were 24 months (95% CI 21–27) and 42 months (40-not estimable [NE]) in the whole population, respectively. By cohort, the median rw-PFS and OS were as follows: 28 (95% CI 23–39) and 44 (95% CI 38-NE) months in patients with *de novo* metastatic disease, 13 (95% CI 11–17) and 36 months (95% CI 31–41) in patients who experienced relapse < 12 months after the end of ET, and 31 months (95% CI 26–37) and not reached (NR) in patients who experienced relapse > 12 months after the end of ET. rw-PFS and OS were longer in patients with oligometastasis and only one metastatic site and those with non-visceral disease. The most frequent hematologic toxicity was neutropenia (72%; grade ≥ 3: 52.5%), and the most common non-hematologic adverse event was asthenia (38%).

**Conclusion:**

These findings, consistent with those from clinical trials, support use of palbociclib plus ET as 1 L for advanced BC in the real-world setting, including pre-menopausal women and men.

**Trial registration number:**

NCT04874025 (PALBOSPAIN). Date of registration: 04/30/2021 retrospectively registered.

**Supplementary Information:**

The online version contains supplementary material available at 10.1007/s10549-024-07287-w.

## Introduction

Endocrine therapy (ET) is the primary treatment option for hormone receptor (HR)-positive and human epidermal growth factor receptor 2 (HER2)-negative advanced breast cancer (ABC) [1, 2]. However, ET is associated with primary or more frequently acquired resistance after exposure to one or more lines of treatment. Loss of cell cycle regulation due to disturbances in the cyclin pathway is common in advanced HR^+^/HER2– breast cancer and has led to development of treatments directed at this target through inhibition of CDK 4/6 [3]. Palbociclib was the first CDK 4/6 inhibitor marketed, and together with the other CDK 4/6 inhibitors (ribociclib and abemaciclib), it represents the greatest therapeutic advance in recent years for treatment of HR^+^/HER2– ABC [4]. The efficacy and safety of palbociclib were demonstrated in the phase 2 clinical trial PALOMA-1 and the two phase 3 trials PALOMA-2 and PALOMA-3 [5–7]. Combination of palbociclib with ET significantly increases progression-free survival (PFS) compared with ET alone as first- and second-line treatment of HR^+^/HER2– ABC. In the PALOMA-2 study, which included post-menopausal women with ABC, addition of palbociclib to letrozole led to a median PFS of 24.8 months compared with 14.5 months with letrozole alone (HR 0.58; 95% CI 0.46–0.72; *p* < 0.00001) [6]. In pre- and post-menopausal patients whose disease progressed during prior endocrine therapy, the PALOMA-3 study showed that the combination of palbociclib and fulvestrant can result in a longer median PFS than fulvestrant alone (9.5 vs. 4.6 months; HR 0.46, 95% CI 0.36–0.59; *p* < 0.0001) [7]. Furthermore, a meta-analysis revealed that combination therapy with CDK 4/6 inhibitors and endocrine therapy is a better therapeutic strategy than endocrine monotherapy for advanced HR^+^/HER2– breast cancer [8].

The benefit of treatment with palbociclib plus hormone therapy has been shown to be independent of age, functional status, sites of metastasis, prior hormone therapy, and progression-free interval since adjuvant therapy [6, 7, 9]. Neutropenia is the most common adverse event (AE) associated with palbociclib treatment. However, it rarely results in permanent discontinuation and is manageable with dose delay and/or reduction [10].

Palbociclib was approved by the Food and Drug Administration (FDA) in 2015 and by the European Medicines Agency (EMA) in 2016 for first-line treatment of patients with HR^+^/HER2– ABC when used in combination with an aromatase inhibitor and in combination with fulvestrant in patients who have received prior hormone therapy [11, 12]. In Spain, palbociclib was marketed in 2017. Although therapy including CDK 4/6 inhibitors is recommended as first-line treatment for HR^+^/HER2– ABC, use of endocrine therapy alone and chemotherapy continues to be significantly high [13]. Overall, results for effectiveness and safety in routine clinical practice may be important to support the recommendation of guidelines [13]. In addition, despite knowledge of palbociclib obtained from controlled clinical trials, real-world studies (RWSs) enable evaluation of its patterns of use in different countries and clinical situations and in determining whether the benefit observed in clinical trials is confirmed in an unselected population. Some RWSs carried out in different populations have already published their results [14–20]. Here, we present PALBOSPAIN, an observational study, to evaluate the effectiveness and safety of palbociclib in clinical practice.

## Methods

### Study design

PALBOSPAIN (NCT04874025) was an observational, retrospective, multicenter study carried out in Spain to evaluate real-world practice patterns and outcomes of first-line treatment with palbociclib in patients with HR^+^/HER2– ABC. The study was performed in accordance with the principles of the Declaration of Helsinki. Approval was granted by the Ethics Committee of the Hospital Clínico San Carlos.

### Study population

The inclusion criteria were as follows: patients diagnosed with breast cancer who started treatment with palbociclib between November 2017 and November 2019, patients with locally advanced or metastatic breast cancer not deemed amenable to curative surgery or curative radiation therapy, patients with HR^+^/HER2– breast cancer, female patients (pre- or post-menopausal) or male patients older than 18 years, patients who received at least one dose of palbociclib, patients who had at least two documented clinical visits after the start of treatment with palbociclib, and patients for whom clinical data were available. For living patients, the ability to understand and sign the informed consent form was also necessary. The exclusion criteria were any previous systemic treatment for advanced disease, treatment with palbociclib conducted in the context of clinical trials or compassionate use programs, HER2^+^ tumor in the most recent or previous biopsy, or HR^−^ tumor in the most recent biopsy.

### Study outcomes

The primary endpoint of this study was real-world progression-free survival (rw-PFS). The secondary endpoints were overall survival (OS), real-world response rate (rw-RR), defined as the percentage of patients who had confirmed complete or partial response, real-world clinical benefit rate (rw-CBR), as defined as having complete response, partial response, or stable disease for at least 24 weeks, dose reduction percentage, and safety.

### Statistical analysis

All patients treated with palbociclib who satisfied all the inclusion criteria and none of the exclusion criteria were included for analysis. Descriptive analysis was performed, and no formal hypothesis was tested. Qualitative variables were presented as measures of central tendency and dispersion (mean [95% CI], median, interquartile range [IQR], minimum and maximum), and quantitative variables were presented using contingency tables.

Survival analyses (rw-PFS, OS) were performed using the Kaplan–Meier method, and comparisons were calculated using the log-rank test. In all survival analyses, periods without events for patients at the time of data cut-off were calculated from the date of treatment start to the date of last follow-up. The association between prognostic factors and survival was examined using the Cox proportional hazards regression model. The Cox regression model is used to analyse the relationship between predictor variables and the time until an event of interest occurs. Prognostic factors considered for the Cox regression model and the subgroup analysis included endocrine sensitivity, age, menopausal status, location and number of metastatic sites and dose received.

## Results

### Study population

From July 2021 to June 2022, 815 patients were screened. Finally, 762 patients from 35 hospitals met the inclusion and exclusion criteria and were included in the analysis (Supplementary Fig. 1). The cut-off date for this analysis was July 2022, and the median duration of follow-up was 29 months (IQR: 21–37). The mean age was 62 years (SD: 12.33). A total of 98.6% of patients were women. Overall, 133 patients were < 50 years (17.5%), 395 patients were 50–70 years (51.8%), and 234 patients were > 79 years (30.7%). A total of 114 patients were pre-menopausal (15%), and 648 were post-menopausal (85%). A total of 418 patients had visceral disease (54.9%). At baseline, 325 patients had 1 metastatic location (42.7%), 328 patients had 2 or 3 metastatic locations (43%), and 109 had more than 3 metastatic locations (14.3%). A total of 127 (16.7%) and 15 (2%) patients were considered to have oligometastatic disease and visceral crisis, respectively, according to ESO-ESMO international consensus guidelines for advanced breast cancer (ABC 5) [2]. Other demographic characteristics of the patients and treatments received are shown in Table 1.

**Table 1:** Clinical characteristics of the patients (*n* = 762)

	Patients, n (%)
Age at initiation of palbociclib (years)	
< 50 50–70 >70	133 (17.5) 395 (51.8) 234 (30.7)
Sex	
Female Male	751 (98.6) 11 (1.4)
Menopausal status	
Pre and perimenopausal Menopausal	117 (15.58) 634 (84.42)
Hormone-receptor status	
ER positive and PR positive ER positive and PR negative	585 (76.77) 163(21.39)
Endocrine sensitivity of breast cancer	
Relapse > 12 months after the end of ET Relapse during the ET or within 12 months of the ET *De novo* metastasis at baseline	289 (37.9) 239 (31.4) 233 (30.6)
Number of metastatic sites	
1 2 or 3 > 3	325 (42.7) 328 (43.0) 109 (14.3)
Metastasis site	
Visceral Non-visceral	418 (54.9) 344 (45.1)
Location of metastasis	
Bone Lung Liver Brain	225 (29.5) 201 (26.4) 163 (21.4) 8 (1.0)
Accompanying endocrine treatment	
• Aromatase inhibitor Relapse during the ET or within 12 months of the ET Relapse > 12 months after the end of ET *De novo* metastasis • Fulvestrant Relapse during the ET or within 12 months of the ET Relapse > 12 months after the end of ET *De novo* metastasis	530 (69.6) 76 (31.8) 240 (83.0) 213 (91.4) 230 (30.2) 161 (67.4) 51 (17.6) 18 (7.7)
Previous treatments for early breast cancer	
Chemotherapy Hormone therapy Radiotherapy	419 (55.0) 485 (63.6) 390 (51.2)
Treatment after disease progression	
Chemotherapy Hormone therapy Targeted therapy	208 (51.7) 188 (47.0) 90 (23.5)

ER: estrogen receptor. ET: adjuvant endocrine therapy. PR: progesterone receptor

### Real-world progression-free survival

**Fig. 1:**
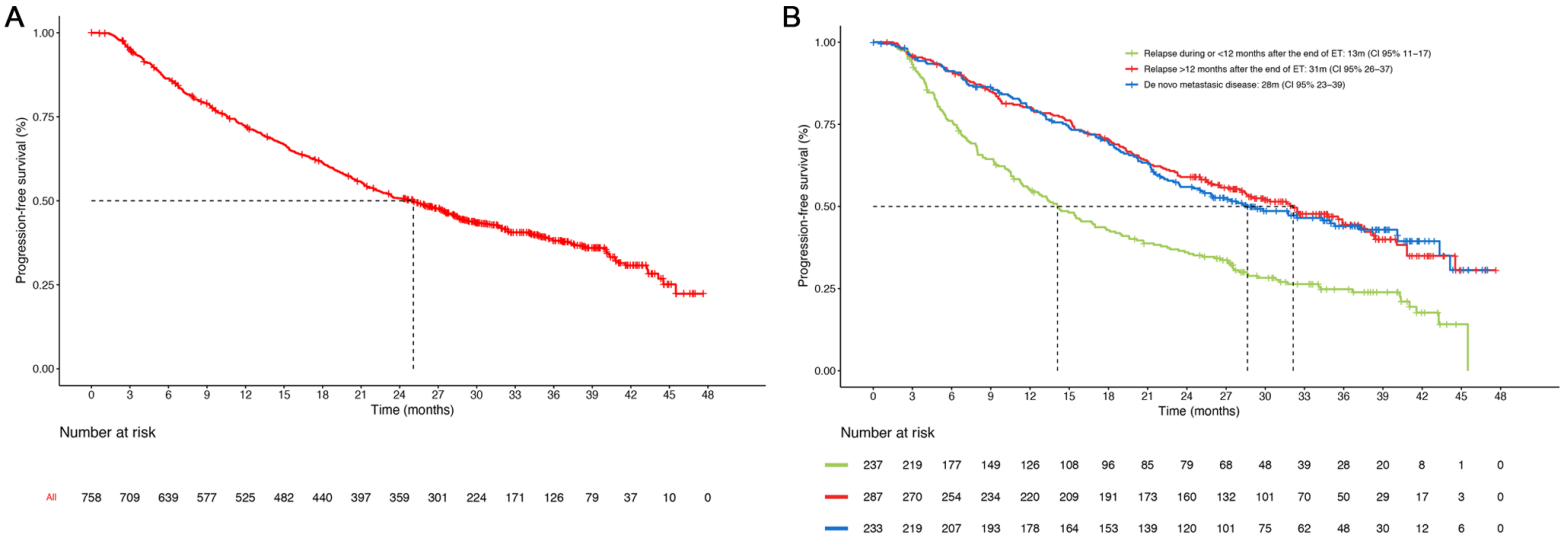
Real-world progression-free survival (rw-PFS) in (**A**) the whole population (*n* = 758) and (**B**) patients with *de novo* metastatic disease, relapse > 12 months after the end of endocrine therapy (ET), and relapse during ET or within 12 months of ET. CI: confidence interval; m: months

**Fig. 2:**
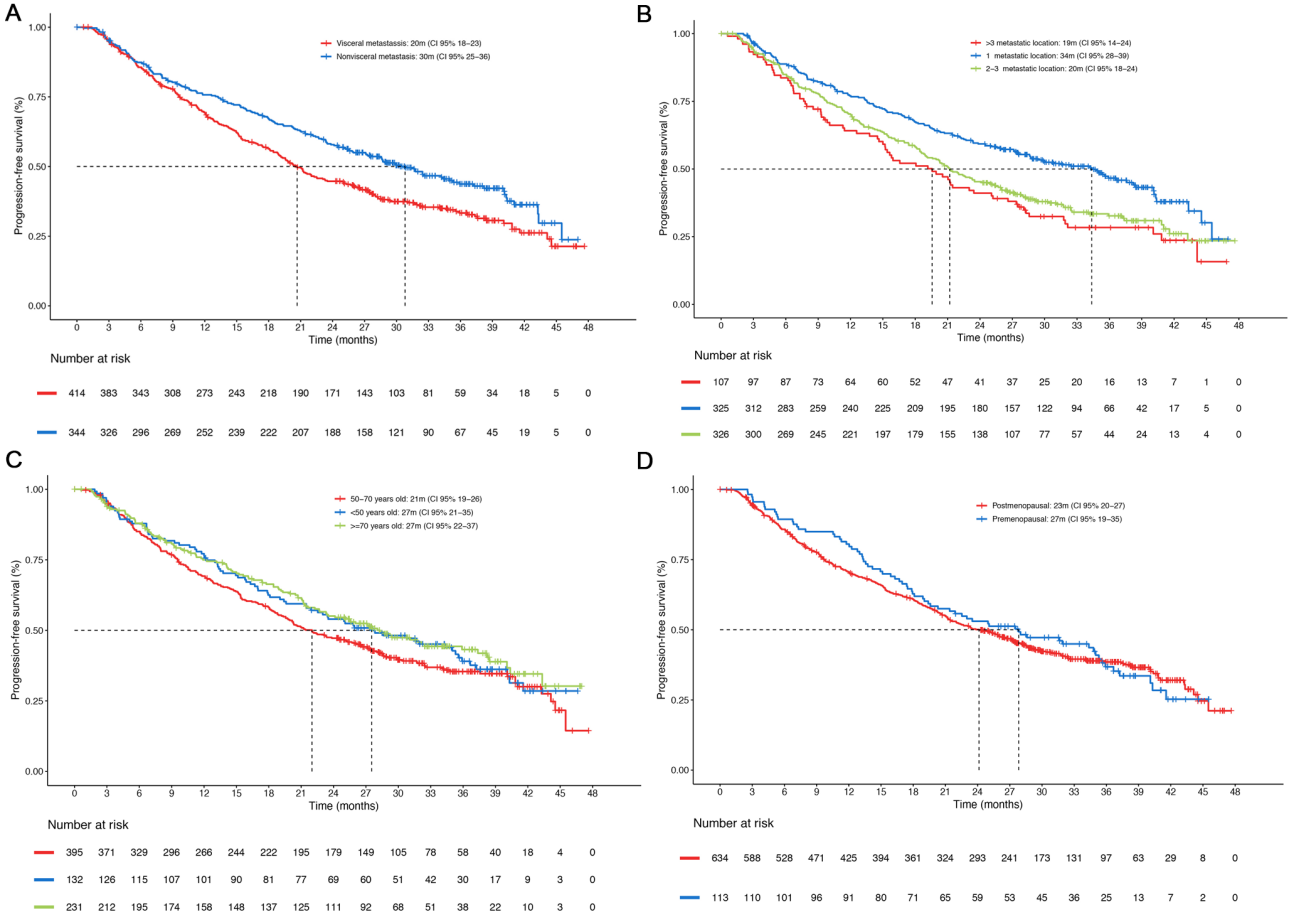
Real-world progression-free survival (rw-PFS) in (**A**) patients with non-visceral vs. visceral metastasis, (**B**) patients with 1, 2 or 3 or more than 3 metastatic sites, (**C**) depending on age, (**D**) depending on menopausal status. CI: Confidence interval; m: months

The median rw-PFS was 24 months (95% CI 21–27) in the whole population. According to previous endocrine therapy exposure, the median rw-PFS was 28 months (95% CI 23–39) in patients with *de novo* metastatic disease (*n* = 233), 31 months (95% CI 26–37) in patients who experienced relapse > 12 months after the end of ET (*n* = 222) and 13 months (95% CI 11–17) in patients who experienced relapse within 12 months of the end of adjuvant ET (*n* = 231) (Fig. 1). There was a significantly longer median rw-PFS in patients with non-visceral vs. visceral metastasis (30 vs. 20 months; HR 0.73; 95% CI 0.60–0.88; *p* = 0.001). Patients with 1 metastatic location had longer rw-PFS than those with > 3 locations (34 vs. 19 months; HR 0.60; 95% CI 0.45-0-79, *p* = 0.0002). The median rw-PFS was similar between patients with 2–3 and > 3 metastatic locations (20 vs. 19 months, respectively; HR 0.87; 95% CI 0.66–1.13, *p* = 0.3) (Fig. 2A and B) but was significantly longer in cases meeting the oligometastatic disease definition vs. non-oligometastatic cases (32 vs. 22 months; HR 0.76; CI 95% 0.59–0.99). There were no significant differences in rw-PFS between different age groups: 27 months in < 50 years old vs. 21 months in 50–70 years old (HR 0.86; 95% CI 0.66–1.18), and 27 months in ≥ 70 years old vs. 21 months in 50–70 years old (HR 0.80; 95% CI 0.65-1.00). No significant differences were observed between menopausal vs. pre-menopausal patients (23 vs. 27 months; HR 0.94; 95% CI 0.73–1.22) (Fig. 2C and D).

### Overall survival

**Fig. 3:**
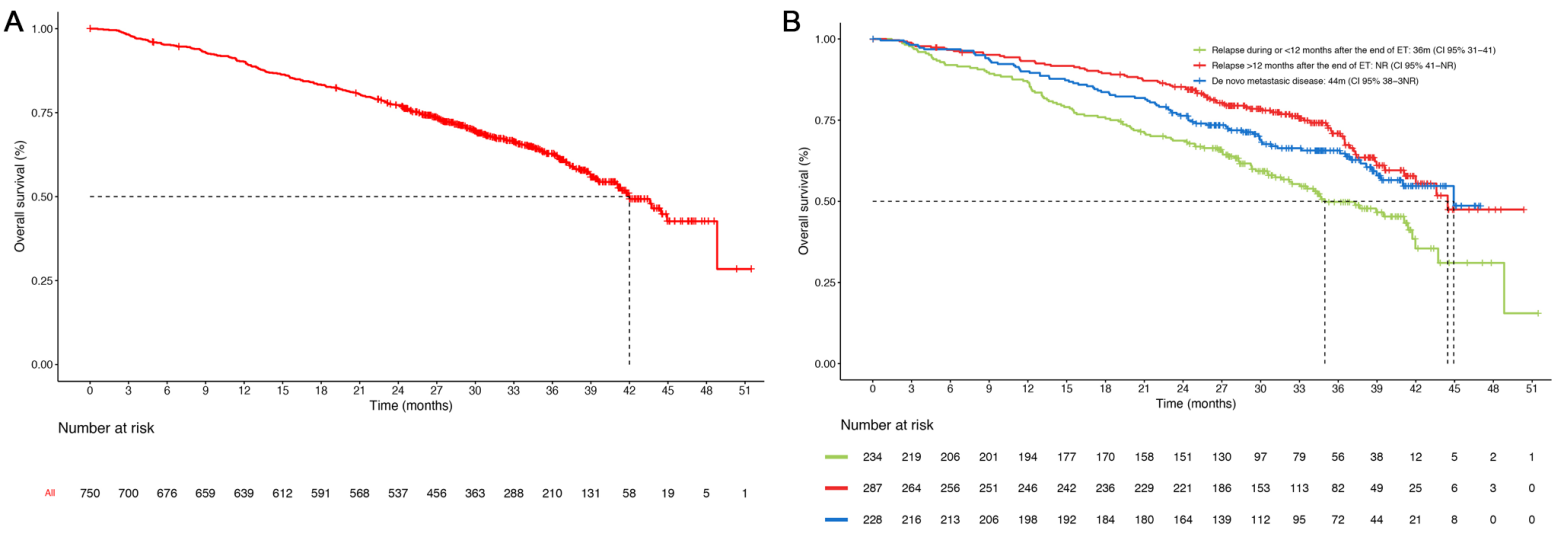
Overall survival in (**A**) the whole population (*n* = 750) and in (**B**) patients with *de novo* metastatic disease, relapse > 12 months after the end of endocrine therapy (ET), and relapse during ET or within 12 months of ET. CI: confidence interval; m: months; NR: not reached

**Fig. 4:**
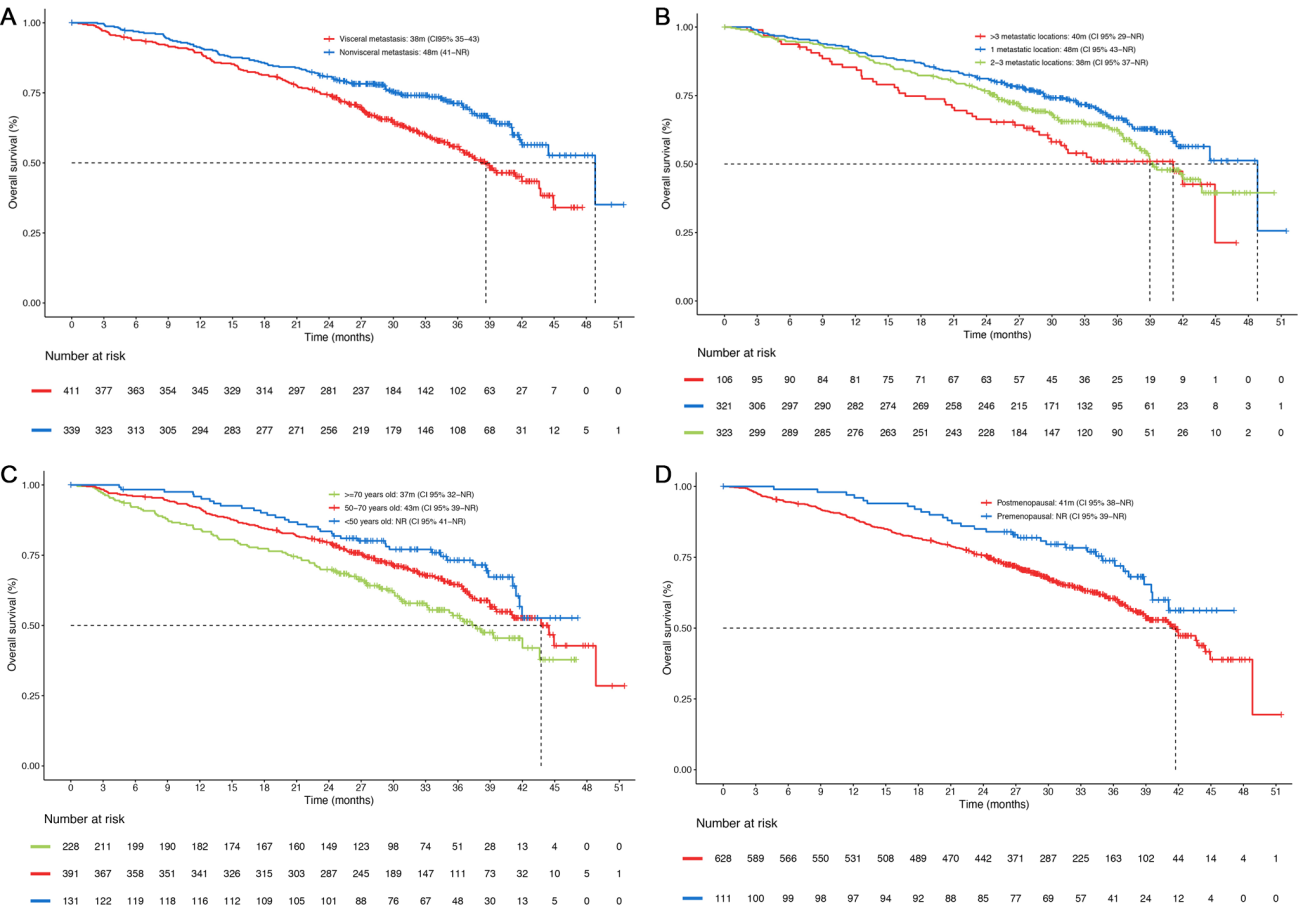
Overall survival (OS) in (**A**) patients with non-visceral vs. visceral metastasis, (**B**) patients with 1, 2 or 3 or more than 3 metastatic sites, (**C**) depending on age, (**D**) depending on menopausal status. CI: Confidence interval; m: months; NR: not reached

The median OS was 42 months (95% CI 40-not estimable [NE]) in the whole population, 44 months (95% CI 38-NE) in the *de novo* metastasis group, 36 months (95% CI 31–41) in patients who experienced relapse ≤ 12 months after the end of adjuvant ET and not reached [NR] in patients who experienced relapse > 12 months after the end of ET) (Fig. 3). There was a significantly longer median OS in patients with non-visceral vs. visceral metastasis (48 vs. 38 months; HR 0.62; 95% CI 0.48–0.80; *p* = 0.0002). Depending on the number of metastatic locations, the median OS was longer in patients with 1 vs. >3 metastatic sites (48 vs. 40 months; HR 0.59; 95% CI 0.42-0-84; *p* = 0.003), but no differences were observed between patients with 2–3 vs. >3 metastatic locations (38 vs. 40 months, respectively; HR 0.79; 95% CI 0.56–1.10, *p* = 0.17) (Fig. 4A and B). Patients with oligometastatic disease had longer overall survival (NR vs. 40 months; HR 0.65; CI 95% 0.46–0.92) than patients without oligometastatic disease, and pre-menopausal patients had longer OS than post-menopausal patients (NR vs. 41 months; HR 0.64; 95% CI 0.44–0.93; *p* = 0.01). Advanced age was associated with shorter survival: the median overall survival in patients > 70 years was 37 months compared to 43 months for patients between 50 and 70 years (HR 0.70; 95% CI 0.54–0.91) and NR for patients < 50 years (HR 0.52; 95% CI 0.35.0.76). (Figure 4C and D).

### Real-world response rate and clinical benefit rate

**Fig. 5:**
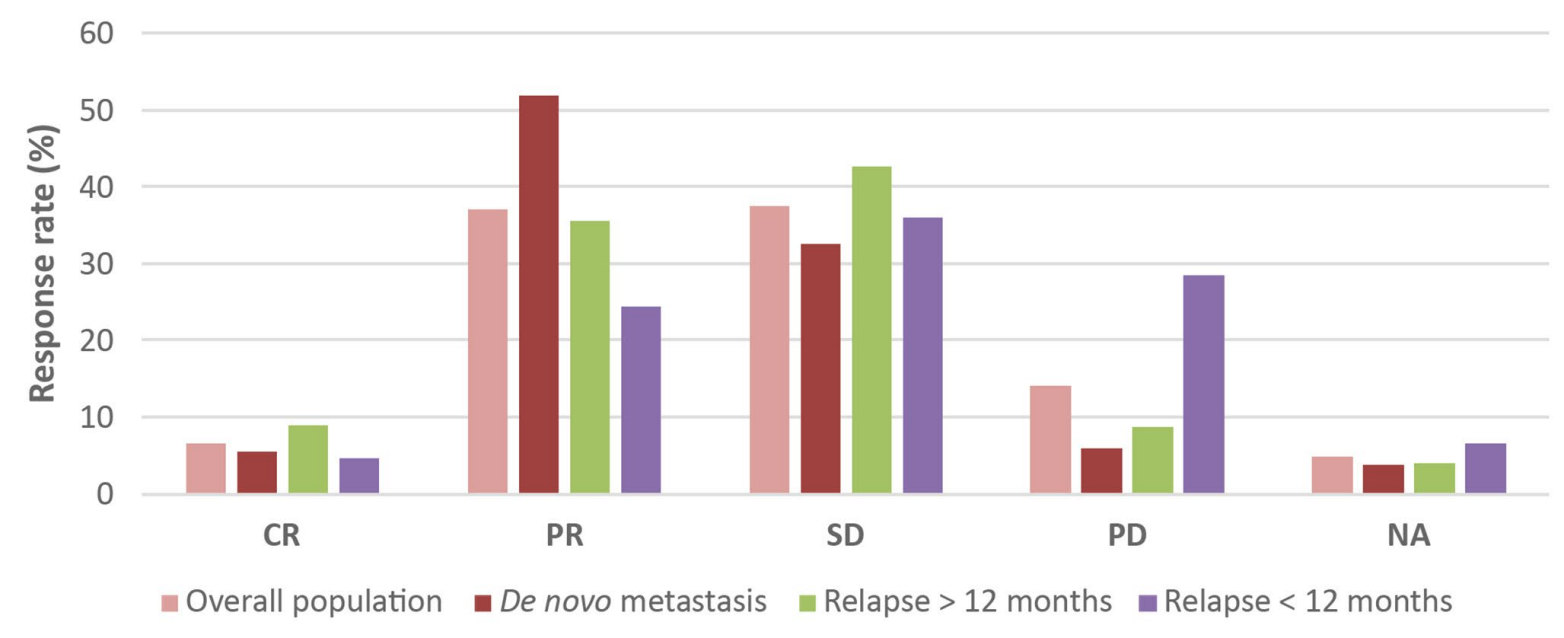
Response rate (rw-RR) in the whole population and in some groups of patients with *de novo* metastasis, relapse > 12 months after the end of the adjuvant endocrine therapy (ET) relapse > 12 months) and relapse during the adjuvant ET or within 12 months of its ending (relapse < 12 months). CR: Complete response; PR: Partial response; SD: Stable disease; PD: Progression of disease; NA: Not assessed

The rw-RR and rw-CBR were 43.6% and 81%, respectively. Overall, 6.6% of patients experienced complete response (CR), 37.0% partial response (PR), 37.5% stable disease (SD), and 14.0% progressive disease. Figure 5 shows the response rate in the whole population and according to previous endocrine treatment. Other results for response are shown in Supplementary Table 1.

### Safety and dose reduction percentages

Hematologic and non-hematologic adverse events were reported in 577 (75.5%) and 412 (54.1%) patients, respectively (Table 2). The most common non-hematologic treatment-related reported events were asthenia (38.8%; grade ≥ 3: 2.1%), arthralgias (19%) and nausea (8%). The most common hematologic toxicity reported was neutropenia (72%; grade ≥ 3: 52.5%), though there were no cases of febrile neutropenia. Three thromboembolic events (all grade 3) and seven pneumonitis events (one of grade 3) occurred.

**Table 2:** Adverse events (*n* = 762)

	All grades, n (%)	Grade ≥ 3, n (%)
Leukopenia	228 (29.9)	39 (5.1)
Neutropenia	549 (72.0)	400 (52.5)
Lymphopenia	102 (13.4)	27 (3.5)
Anemia	223 (29.3)	23 (3.0)
Thrombocytopenia	129 (16.9)	17 (2.2)
Nausea	61 (8.0)	0 (0.0)
Vomiting	27 (3.5)	2 (0.3)
Pyrosis	17 (2.2)	0 (0.0)
Asthenia	296 (38.8)	16 (2.1)
Rash	32 (4.2)	1 (0.1)
Alopecia	60 (7.9)	2 (0.2)
Pneumonitis	7 (0.9)	1 (0.1)
Arthralgia	145 (19.0)	1 (0.1)
ALT/AST increase	61 (8.0)	10 (1.3)

A total of 385 patients (50.5%) underwent a dose reduction of palbociclib; 56.5% of them had one dose reduction, and 43.5% had two dose reductions. The final dose administered was 100 mg for 213 patients (27.9%) and 75 mg for 164 (21.6%).

## Discussion

Pivotal clinical trials and a meta-analysis have shown that the combination of CDK 4/6 inhibitors with ET prolongs survival in patients with advanced HR^+^/HER2– breast cancer [21–24]. Following approval of CDK 4/6 inhibitors, several real-world evidence studies have been carried out to complement randomized clinical trial data. Data from real-world and clinical trial outcomes for CDK 4/6 inhibitors can provide a comprehensive picture to inform decisions about clinical treatment. PALBOSPAIN is the largest European RWS of palbociclib used as first-line treatment for advanced HR^+^/HER2– breast cancer (762 patients).

In PALBOSPAIN, the median rw-PFS (24.0 months, 95% CI 21–27) in the whole population was similar to that found in patients treated with palbociclib plus ET in the PALOMA-2 study (24.8 months, 95% CI 22.1-NE) [6]. Recently, a large Danish RWS that included 728 patients in the first-line setting reported a rw-PFS of 24.3 months (95% CI 21.7–27.8) [14]. In other RWSs using palbociclib as first-line therapy, PFS ranged between 15.1 and 26.4 months [15–17]. In the largest RWS published thus far, P-REALITY X assessed outcomes for 2,888 post-menopausal women and men with HR^+^/HER2^−^ ABC who had received first-line treatment with either palbociclib and an aromatase inhibitor (AI) or an AI alone. Patients who received palbociclib had a significantly longer rw-PFS (19.3 months versus 13.9 months; HR 0.70, 95% CI 0.62 to 0.78, *p* < 0.0001) and OS (49.1 months versus 43.2 months; HR 0.76, 95% CI 0.65 to 0.87, *p* < 0.0001) [18].

As shown in the PALOMA 2 & 3 trials, endocrine sensitivity is a significant prognostic factor for survival in patients with ER+/HER2- ABC treated with palbociclib [6, 7]. In PALBOSPAIN, the median rw-PFS of patients with *de novo* metastatic disease (30.6%), who experienced relapse > 12 months (37.9%) or who experienced relapse < 12 months after completion of adjuvant ET (31.4%) were 28, 31 and 13 months, respectively. In the PALOMA-2 study, the median PFS found in patients with *de novo* metastatic disease and patients with relapse > 12 months was the same (27.9 and 27.6 months, respectively), and 16.6 months in patients with relapse < 12 months [25]. Progression-free survival in the cohort of endocrine-sensitive patients (*de novo* metastatic and disease-free interval [DFI] > 12 months) was similar to that observed in the PALOMA-2 study; in those with endocrine-resistant disease (DFI ≤ 12 months), the median progression-free survival was similar to that achieved with fulvestrant + palbociclib in the PALOMA-3 study (9.5 months: 95% CI 7.4-NE) [7].

Although in clinical trials patients with *de novo* metastatic disease and those with late relapses are usually included within the hormone-sensitive (endocrine-sensitive) population, the biological behaviour and sensitivity to hormonal treatment may be different, given that patients with late relapses have shown a sensitivity to hormonal treatment while patients with *de novo* metastatic disease may present primary resistance to endocrine treatment, and this could explain the results observed in these patients in terms of PFS (shorter rw-PFS in patients with *de novo* disease [28 months] than in patients who experienced relapse > 12 months after the end of ET [31 months]) and OS.

The overall survival of the patients included in the PALBOSPAIN study was lower than that described in the PALOMA-2 study (42 vs. 53.9 months, respectively) [6], in which palbociclib plus letrozole did not improve OS vs. placebo plus letrozole (53.9 vs. 51.2 months; HR 0.95; *p* = 0.33). However, interpretation of OS in the PALOMA-2 trial is limited by the extensive and disproportionate censoring of patients with missing survival data [26]. Most probably, the inclusion of a higher percentage of patients with recurrences during adjuvant hormonal treatment (31.4% vs. 22.3%) contributed to differences between our results and those of the PALOMA-2 study. As previously shown, sensitivity to prior endocrine therapy has a significant influence on overall survival. In fact, in the PALOMA-2 study, the median OS in patients with DFI > 12 months after completion of adjuvant hormonal therapy was 66.3 months (95% CI 52.1–79.7) in the palbociclib plus letrozole group. More recent results from the PALOMA-2 study show an OS of 54.6 months in patients with *de novo* metastatic disease and of 66.3 months in patients with DFI > 12 months [26]. In the PALBOSPAIN study, the median OS was not reached in the group of patients with DFI > 12 months, whereas it was 36 months (95% CI 31–41) in patients with DFI ≤ 12 months, similar to that observed in the PALOMA-3 trial (34.8 months; 95% CI 28.8–39.9) [7]. Similarly, the median OS in the P-REALITY X study was longer than that in the PALBOSPAIN study (49.1 vs. 42 months, respectively) [18]. Although the P-REALITY X study did not specify the percentage of patients based on sensitivity to endocrine treatment, patients who experienced relapse in the first 5 years after diagnosis only represented 21.8% of the total population, which suggests that it is a more hormone-sensitive population than that described in our study.

The location of metastatic disease can influence treatment outcomes and prognosis. Subgroup analyses of the PALOMA 2 & 3 trials indicated that the benefits of palbociclib were consistent regardless of the presence of visceral metastasis. We found significantly longer rw-PFS (30 vs. 20 months¸ *p* = 0.001) and OS (48 vs. 38 months; *p* = 0.0002) in patients with non-visceral vs. visceral metastasis. Similar results have been reported by Garly R et al. in patients treated with palbociclib as first- and second-line therapy (median rw-PFS and OS of 16.6 and 36.6 months, respectively) [14]. In addition, the number of metastatic sites can affect the overall tumor burden and aggressiveness of the disease, potentially impacting response to treatment. PALOMA clinical trials did not specifically analyse outcomes based on the number of metastatic sites. In the PALBOSPAIN study, we observed a significant improvement in rw-PFS and OS in patients with 1 metastatic location vs. those with ≥ 2 sites of metastasis.

Older patients are usually underrepresented in clinical trials. In fact, only 48 and 27 patients aged ≥ 75 years treated with palbociclib were included in the PALOMA 2 and 3 trials, respectively. With an increasing number of older patients expected to develop breast cancer in the coming years, understanding the safety and efficacy of anticancer treatments for older patients should be a clinical and research priority. A total of 228 patients aged ≥ 70 years were included in the PALBOSPAIN study. Although the median rw-PFS was similar to that of younger age groups, as previously described by Clifton et al., the median OS was shorter than that of younger patients [19]. This difference may be related to the lower life expectancy and existence of competing causes of death in older women. However, in the P-REALITY X study, improvements in PFS and OS were observed regardless of age; thus, older women should not be excluded from CDK 4/6 inhibitor treatment.

The MONALEESA-7 trial that studied ribociclib plus ET vs. placebo plus ET is the only phase III trial dedicated specifically to pre- and perimenopausal women with HR+/HER2- ABC. Overall survival was significantly longer in the ribociclib group than in the placebo group, with a 29% lower risk of death (hazard ratio for death, 0.71; 95% CI, 0.54 to 0.95) [22]. In the PALBOSPAIN study, we observed a similar median rw-PFS in pre- and post-menopausal patients. Interestingly, the median OS was longer in pre- vs. post-menopausal patients (NR vs. 41 months; HR 0.64; 95% CI 0.44–0.93; *p* = 0.01). Although pre-menopausal women were not included in the PALOMA-2 [6] study, the results of PALBOSPAIN support use of palbociclib plus ET as an option in first-line therapy for pre-menopausal women with HR^+^HER2– ABC.

Patients in the PALBOSPAIN study and the group of patients treated with palbociclib-letrozole in the PALOMA-2 study [6] showed very similar response rw-RRs (43.6% and 42.1%, respectively) and rw-CBRs (81.0% vs. 84.9%, respectively). The response rate was higher in the most endocrine-sensitive cohorts (*de novo* metastasis and DFI > 12 months) than in the endocrine-resistant cohorts (DFI ≤ 12 months). These results are consistent with those observed in the PALOMA-1 study (RR 43% and CBR of 81% in patients treated with palbociclib-letrozole) and in other RWSs (rw-RR and rw-CBR ranging from 68.3 to 84.5% and 88–97.5%, respectively) [5, 20, 27].

Regarding safety, for non-hematologic adverse effects, the frequency of asthenia was similar in PALBOSPAIN (38.8%) and PALOMA-2 (37.4%), but gastrointestinal toxicity was less frequent in PALBOSPAIN than in PALOMA-2 (nausea 8% vs. 35.1%; vomiting 3.5% vs. 15.5%), as were rash (4.2% vs. 17.8%) and arthralgia (19% vs. 33.2%) [6]. The hematological adverse effects observed in PALBOSPAIN were consistent with those reported in clinical trials and in RWSs, with neutropenia being the most frequent (72%). However, the percentage of grade ≥ 3 neutropenia (52.5%) was lower than that reported in clinical trials (54% in PALOMA-1, 66.4% in PALOMA-2, 65% in PALOMA-3 [5–7]) and similar to that in other RWSs, ranging between 41.5% and 63% [15–17, 28–33]. The lower percentage of grade ≥ 3 neutropenia in the PALBOSPAIN study (54%) than in clinical trials might be related to a higher frequency of palbociclib dose reduction in PALBOSPAIN than in PALOMA-2 and PALOMA-3, as well as to less exhaustive monitoring of hematological toxicity and recording of adverse events in routine clinical practice than in clinical trials.

Adverse events considered of special interest (pneumonitis and thromboembolic events) occurred in < 1% of patients included in PALBOSPAIN. In a retrospective study, Watson GA et al. [34] reported a high incidence of thromboembolic events (11%), a significant increase compared with the 2% found in the PALOMA-3 trial [7]. However, the tolerability profile was considered manageable, without any detrimental impact on quality of life [35]. In fact, a survey of 604 patients in six countries showed that 96% of patients treated with palbociclib reported high satisfaction scores [36].

The main strengths of this study are the high number of patients included and the presence of a heterogeneous real-world population (pre-menopausal and male patients, as well as patients with comorbidities and visceral crisis) not commonly represented in clinical trials. However, the PALBOSPAIN study has some limitations. As in other RWSs, there is potential for missing, inaccurate, or incomplete data. Only data from centers willing and able to participate in this study were collected, and when selecting a sample of patients, investigators introduced selection biases. Consequently, the characteristics of the patients in this sample may be different from those of ABC patients in other Spanish centers, and the results may not be externally generalizable. Finally, the schedule and method of tumor assessments were dictated by the treating physician and do not necessarily adhere to RECIST criteria; hence, the results from this real-world study may not be directly comparable to those from clinical trials.

## Conclusions

The median rw-PFS, rw-RR and OS in PALBOSPAIN were consistent with the palbociclib efficacy shown in PALOMA-2. Dose reduction was more frequent in the PALBOSPAIN trial than in the PALOMA-2 and PALOMA-3 trials, though without a negative impact on efficacy. The safety profile corresponded to previously published results and indicated that palbociclib has manageable tolerability in daily clinical practice. This study’s findings about effectiveness and safety offer information complementary to that obtained from pivotal clinical trials.

### Electronic supplementary material

Below is the link to the electronic supplementary material.

Supplementary Material 1

## Abbreviations

1 L: First line
ABC: Advanced breast cancer
AE: Adverse event
AI: Aromatase inhibitor
BC: Breast cancer
CBR: Clinical benefit rate
CDK: Cyclin-dependent kinase
CI: Confidence interval
CR: Complete response
DFI: Disease-free interval
EMA: European Medicines Agency
ESO-ESMO: European School of Oncology - European Society for Medical Oncology
ET: Endocrine therapy
FDA: Food and Drug Administration
HR: Hazard ratio
HR^+^/HER2^−^: Hormone receptor (HR) positive and human epidermal growth factor receptor 2 (HER2) negative
IQR: Interquartile range
NA: Not assessed
NE: Not estimable
NR: Not reached
OS: Overall survival
PD: Progression of disease
PFS: Progression-free survival
PR: Progesterone receptor
PR: Partial response
RECIST: Response Evaluation Criteria in Solid Tumors
rw-CBR: Real-world clinical benefit rate
rw-RR: Real-world response rate
rw-PFS: Real-world progression-free survival
RWS: Real-world study
rw-RR: Real-world response rate
SD: Standard deviation
SD: Stable disease
