# Supplementary material for: First-line therapy with palbociclib in patients with advanced HR+/HER2− breast cancer: The real-life study PALBOSPAIN
Source: Breast Cancer Res Treat. 2024 Apr 1;206(2):317–28. doi: 10.1007/s10549-024-07287-w (PMC11182794; doi:10.1007/s10549-024-07287-w)
Supplement: Supplementary file 1 — Supplementary Material 1 [file 10549_2024_7287_MOESM1_ESM.docx]

**Supplementary Table 1.** Response rate (rw-RR) in several groups of patients

|  | **Complete response**  **n (%)** | **Partial response**  **n (%)** | **Stable disease**  **n (%)** | **Disease progression**  **n (%)** | **Not assessed**  **n (%)** |
| --- | --- | --- | --- | --- | --- |
| Pre-menopausal patients  Post-menopausal patients | 13 (11.5)  37 (5.8) | 43 (38.1)  234 (36.7) | 40 (35.4)  241 (37.8) | 15 (13.3)  91 (14.3) | 2 (5.4)  35 (5.5) |
| Visceral metastasis  Non-visceral metastasis | 21 (5.0)  29 (8.4) | 173 (41.4)  109 (31.7) | 141 (33.7)  145 (42.2) | 63 (15.1)  44 (12.8) | 20 (4.8)  17 (4.9) |
| Age  <50  50-70  ≥70 | 15 (11.3)  22 (5.6)  13 (5.6) | 50 (37.6)  145 (36.7)  87 (37.2) | 46 (34.6)  152 (38.5)  88 (37.6) | 19 (14.3)  57 (14.4)  31 (13.2) | 3 (2.3)  19 (4.8)  15 (6.4) |
| Number of metastatic sites  1  2 or 3  More than 3 | 24 (7.4)  23 (7.0)  3 (2.8) | 100 (30.8)  132 (40.2)  50 (45.9) | 136 (41.8)  116 (35.4)  34 (31.2) | 46 (14.2)  47 (14.3)  14 (12.8) | 19 (5.8)  10 (3.0)  8 (7.3) |


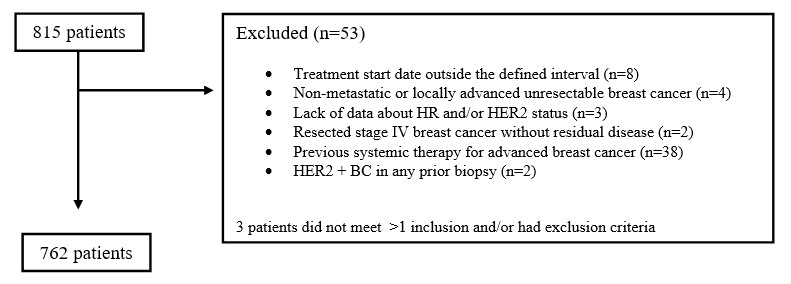


**Supplementary Figure 1** Flowchart of the PALBOSPAIN study

BC: breast cancer; HR: hormone receptor; HER2: human epidermal growth factor receptor 2
